# Cocaine Directly Inhibits α6-Containing Nicotinic Acetylcholine Receptors in Human SH-EP1 Cells and Mouse VTA DA Neurons

**DOI:** 10.3389/fphar.2019.00072

**Published:** 2019-02-14

**Authors:** Dejie Chen, Fenfei Gao, Xiaokuang Ma, Jason Brek Eaton, Yuanbing Huang, Ming Gao, Yongchang Chang, Zegang Ma, Taleen Der-Ghazarian, Janet Neisewander, Paul Whiteaker, Jie Wu, Quanxi Su

**Affiliations:** ^1^ Department of Neurology, Yunfu People’s Hospital, Yunfu, China; ^2^ Division of Neurobiology, Barrow Neurological Institute, St. Joseph’s Hospital and Medical Center, Phoenix, AZ, United States; ^3^ Department of Pharmacology, Shantou University Medical College, Shantou, China; ^4^ Department of Physiology, Qingdao University of Medical College, Qingdao, China; ^5^ School of Life Sciences, Arizona State University, Tempe, AZ, United States

**Keywords:** cocaine, nicotinic acetylcholine receptor, SH-EP1 cells, VTA, dopamine neurons, patch clamp

## Abstract

Alpha6-containing nicotinic acetylcholine receptors are primarily found in neurons of the midbrain dopaminergic (DA) system, suggesting these receptors are potentially involved in drug reward and dependence. Here, we report a novel effect that cocaine directly inhibits α6N/α3Cβ2β3-nAChR (α6*-nAChRs) function. Human α6*-nAChRs were heterologously expressed within cells of the SH-EP1 cell line for functional characterization. Mechanically dissociated DA neurons from mouse ventral tegmental area (VTA) were used as a model of presynaptic α6*-nAChR activation since this method preserves terminal boutons. Patch-clamp recordings in whole-cell configuration were used to measure α6*-nAChR function as well as evaluate the effects of cocaine. In SH-EP1 cells containing heterologously expressed human α6*-nAChRs, cocaine inhibits nicotine-induced inward currents in a concentration-dependent manner with an IC_50_ value of 30 μM. Interestingly, in the presence of 30 μM cocaine, the maximal current response of the nicotine concentration-response curve is reduced without changing nicotine’s EC_50_ value, suggesting a noncompetitive mechanism. Furthermore, analysis of whole-cell current kinetics demonstrated that cocaine slows nAChR channel activation but accelerates whole-cell current decay time. Our findings demonstrate that cocaine-induced inhibition occurs solely with bath application, but not during intracellular administration, and this inhibition is not use-dependent. Additionally, in *Xenopus* oocytes, cocaine inhibits both α6N/α3Cβ2β3-nAChRs and α6M211L/α3ICβ2β3-nCAhRs similarly, suggesting that cocaine may not act on the α3 transmembrane domain of chimeric α6N/α3Cβ2β3-nAChR. In mechanically isolated VTA DA neurons, cocaine abolishes α6*-nAChR-mediated enhancement of spontaneous inhibitory postsynaptic currents (sIPSCs). Collectively, these studies provide the first evidence that cocaine directly inhibits the function of both heterologously and naturally expressed α6*-nAChRs. These findings suggest that α6*-nAChRs may provide a novel pharmacological target mediating the effects of cocaine and may underlie a novel mechanism of cocaine reward and dependence.

## Introduction

Cocaine is a widely used psychostimulant drug, and its abuse is associated with multiple physical and psychiatric sequelae. Cocaine addiction is a serious public health problem worldwide, leading to alterations in memory, behavior, and neuronal physiology. The first clinical signs of cocaine toxicity are typically palpitations, epistaxis, sweating, headache, anxiety, tremors, muscle spasm, and hyperventilation (Nnadi et al., 2005). In humans, neuropsychiatric complications are evident in approximately 60% of cocaine users including depression, suicidal ideation, paranoia, kleptomania, and catatonia. Cocaine use disorder also leads to a series of neurological disorders such as seizures, optic neuropathy, cerebral infarction, subarachnoid and intracerebral hemorrhage, multifocal cerebral ischemia, and cerebral atrophy, as well as cardiac dysfunction including myocardial infarction triggering global brain ischemia and edema (Moorman et al., 2013; Bodmer et al., 2014; Cagienard et al., 2014; Sharma et al., 2014; Sordo et al., 2014). Despite this broad foundation of clinical knowledge, the detailed mechanisms underlying cocaine-induced effects in the brain remain largely unknown.

Previous investigations have established that cocaine increases inter-synaptic dopamine levels through inhibition of dopamine transporters, which produces its rewarding effect (Ritz et al., 1987; Jones et al., 1995); however, cocaine also acts on other targets and influences midbrain dopamine (DA) neuronal activity and DA release. For example, *in vivo* electrophysiological recordings showed that acute intravenous administration of cocaine caused a significant, dose-dependent, partial inhibition (50–70%) of the firing of antidromically identified mesoaccumbens DA neurons, and both somatodendritic impulse-regulating DA autoreceptors (D2) and inhibitory nucleus accumbens-ventral tegmental area (NAc-VTA) feedback processes are involved in the effects (Einhorn et al., 1988). With longer time course (after injection 24 h), single cocaine injection (i.p.) increases the firing rate and bursting activity of VTA dopamine neurons, and these increases persist for 7 days (Creed et al., 2016). In addition, during cocaine withdrawal, there is a behavioral depression that is associated with decreased spontaneous activity of VTA dopamine neurons (Koeltzow and White, 2003). Pharmacological effects of cocaine on VTA DA neuronal function have been shown by a single, systemic administration of cocaine to a mouse or a rat, which affects excitatory synaptic transmission onto DA neurons for days (Saal et al., 2003). Cocaine also modulates meso-limbofrontal neurons through an intrinsic mechanism including that cocaine repeated exposure increases voltage-sensitive calcium currents in response to membrane depolarization in medial prefrontal cortex pyramidal neurons (Nasif et al., 2005), repeated cocaine treatment decreases whole-cell calcium current in rat NAc neurons (Zhang et al., 2002), and cocaine withdrawal reduces sodium currents in NAc neurons (Zhang et al., 1998). Collectively, cocaine exhibits very complex effects on meso-limbofrontal system through modulations of DA neuronal function and DA release, which may underlie cocaine-induced behavioral changes.

VTA neurons express a variety of nicotinic acetylcholine receptor (nAChR) subtypes including α4β2, α7, and α6*-nAChRs, and activation/desensitization of these nAChRs alters VTA DA neuronal activity and DA release (Klink et al., 2001; Azam et al., 2002; Drenan et al., 2008; Yang et al., 2009a, 2011; Wang et al., 2014). In laboratory animals, stimulation of nAChRs by nicotine (NIC) increases cocaine-induced locomotor sensitization (Schoffelmeer et al., 2002) and also produces long-term increases in both locomotor activity and cocaine self-administration in adolescent but not adult rats (Reed and Izenwasser, 2017). While a nonselective nAChR antagonist such as mecamylamine reduced cocaine’s reinforcement in rats (Blokhina et al., 2005), local injection of a selective β2*-nAChR antagonist (dihydro-beta-erythroidine, DHβE) into the VTA prevents cocaine-induced locomotor activity (Champtiaux et al., 2006). Pretreatment with nicotine reduces cocaine-conditioned place preference (CPP) established in rats, but inhibition of nAChRs with mecamylamine also slightly attenuates cocaine-induced CPP in rats (Zachariou et al., 2001; Sershen et al., 2010; Levine et al., 2011). Recently, it has been reported that α4β2 nicotinic receptor desensitizing compounds can decrease the self-administration of cocaine and methamphetamine in rats (Levin et al., 2018). In addition to modulating cocaine-related behavior, differential nicotinic antagonists perfused into the NAc or the VTA also regulate cocaine-induced dopamine release in the NAc of mice (Zanetti et al., 2007). In monkey cocaine self-administration model, the combination of marginally reinforcing doses of cocaine and nicotine increased drug self-administration behavior above levels observed with the same dose of either cocaine or nicotine alone (Mello and Newman, 2011). An α4β2-nAChR partial agonist, varenicline-induced reduction on nicotine+cocaine combinations is dependent on the dose of cocaine (Mello et al., 2014) although varenicline attenuates the reinforcing effects of nicotine alone but not cocaine alone (Gould et al., 2011; Mello et al., 2014). Considering varenicline is an α4β2-nAChR partial agonist and an α7-nAChR full agonist, the above data suggest that partial activation of α4β2-nAChRs and/or full activation of α7-nAChRs may not play a critical role in the modulations of cocaine self-administration in monkey. Therefore, in this study, we focus on examination of the effects of cocaine on α6-containing nAChRs.

Accumulating lines of evidence demonstrate that cocaine inhibits heterologously expressed nAChR subtypes in *Xenopus* oocytes (Damaj et al., 1999; Francis et al., 2000). Cocaine also inhibits a natural nAChR-mediated current in VTA DA neurons and, consequently, increases the ratio of phasic to tonic DA release, thus potentially enhances its reinforcing effects (Acevedo-Rodriguez et al., 2014). Additionally, blockade of nAChRs may mediate cocaine craving and addictive effects (Williams and Adinoff, 2008; Crunelle et al., 2010). These lines of evidence suggest an important interaction between cocaine and nAChRs necessitating further investigation. Interestingly, cocaine-induced CPP is entirely absent in nAChR α6 subunit knockout (KO) mice (Sanjakdar et al., 2015). The VTA expresses multiple nAChR subtypes (Yang et al., 2009a; Wu and Lukas, 2011), but the nAChR α6 subunit is expressed at the highest concentrations in neurons of the midbrain dopaminergic system, including the VTA and substantia nigra pars compacta (SNc) (Xiao et al., 2009; Yang et al., 2009b, 2011), thus midbrain α6*-nAChRs may play a role in cocaine reward and dependence (Azam et al., 2002; Yang et al., 2009b; Acevedo-Rodriguez et al., 2014). However, it is still unknown whether cocaine modulates α6*-nAChRs directly, which is the question this study is designed to address.

In the present study, we evaluated the acute effects of cocaine on α6*-nAChRs heterologously expressed in human epithelial cells using patch-clamp whole-cell recording and found that cocaine noncompetitively inhibits α6*-nAChR-mediated transmembrane current. Additionally, in mechanically dissociated VTA GABA neurons, 1 μM nicotine enhanced spontaneous inhibitory postsynaptic current (sIPSC) frequency, which was sensitive to an α6*-nAChR-selective antagonist (α-conotoxin MII, α-Ctx MII, 10 nM). This suggests that the nicotine-activated natural α6*-nAChRs are located on GABAergic presynaptic boutons of VTA GABA neurons as we recently described (Steffensen et al., 2018). Interestingly, cocaine (10 μM) prevented this nicotine-induced enhancement of sIPSCs, suggesting that cocaine also inhibits natural α6*-nAChRs. Collectively, our study provides the first evidence that cocaine directly inhibits both heterologously expressed and natural α6*-nAChRs, suggesting that α6*-nAChR is likely a cocaine target which may be involved in the process of cocaine reward and dependence.

## Materials and Methods

This study was carried out in accordance with the recommendations of the National Research Council’s Guide for the Care and Use of Laboratory Animals. The protocol was approved by the Barrow Neurological Institute’s Institutional Animal Care and Use Committee guidelines.

### Expression of Human Neuronal α6/α3β2β3-nAChR in Human SH-EP1 Cells

Construction of the human epithelial cell line expressing α6/α3chimeraα2α3-nAChR (α6N/α3Cα2α3-nAChR) was developed by Dr. Whiteaker’s laboratory (Breining et al., 2012). The α6/α3 denotes a chimeric subunit composed of the extracellular, ligand-binding domain of the human α6 subunit fused to the first transmembrane domain and following the sequence of the human α3-nAChR subunit. This approach reproducibly increases expression compared to levels observed with native α6 subunits while retaining α6-like pharmacology (Kuryatov et al., 2000). Consensus-sequence α2 and α3 human nAChR subunit clones were also used. To summarize the salient details, wild-type SH-EP1 cells were transfected with nAChR subunit clones using the cationic polymer Effectene (Qiagen, Valencia, CA). The detailed description of expression procedures and functional and pharmacological properties can be found in our current publication (Chen et al., 2018).

### Preparation of Mechanically Dissociated VTA Neurons

Neurons with functional terminals were obtained by mechanical dissociation as described previously (Akaike and Moorhouse, 2003; Yang et al., 2011; Steffensen et al., 2018). In brief, one midbrain slice was transferred to a 35-mm culture dish (Falcon, Rutherford, NJ) filled with a standard external solution. The region of the slice containing the VTA was directly visualized through an inverted microscope (Nikon, Tokyo, Japan). A fire-polished glass pipette with a 50-μm diameter tip was mounted to a custom-constructed mechanoelectrical device for cellular dissociation. Using a manipulator, the pipette was then positioned just below the liquid-tissue interface of the VTA region. Neurons close to the surface of the slice were dissociated by horizontal vibration at a frequency of 15–20 Hz with a range from 0.1 to 0.3 mm for 2 min. The slice was then removed from the solution containing the dissociated cells. Within 20 min, the isolated neurons adhered to the bottom of the dish and were ready for electrophysiological recordings. These mechanically dissociated neurons differ from neurons dissociated using enzymatic techniques, with the latter losing most, if not all, presynaptic terminals during the dissociation process, the former can, in contrast, retain functional nerve terminals following this process.

### Patch-Clamp Whole-Cell Current Recordings From Transfected α6-nAChRs in SH-EP1 Cells

Standard whole-cell current recordings using a perfusion system allowing fast application and removal of drugs were performed as previously described (Wu et al., 2004, 2006; Yu et al., 2009). Briefly, transfected SH-EP1 cells were prepared in 35-mm culture dishes and then plated on the bottom of the dishes and later placed on the stage of an inverted microscope (IX70 Narishige, Japan). Glass microelectrodes with 3–5 MΩ resistance between the pipette and extracellular solution were used to form tight seals (>1GΩ) on the surface of the cells. The standard whole-cell current recording protocol was initiated by applying a gentle suction to the pipette and continued for 5–10 min to allow for the exchange of the pipette solution and the cytosol to stabilize. Subsequently, recorded cells were gently lifted from the bottom of the culture dishes, which allowed for improved solution exchange and a more accurate evaluation of differences in the kinetics of agonist-induced whole-cell currents. Prior to capacitance and resistance compensation, access resistance (Ra) was measured and deemed acceptable for continuation if observed to be lower than 20 MΩ. Pipette and whole-current capacitance were both minimized, and series resistance was routinely compensated to 80%. Recorded cells were voltage-clamped at a holding potential of −40 mV, and inward currents induced by nicotine were measured (Axopatch 200B amplifier; Molecular Devices, Sunnyvale, CA). Current signals were typically filtered at 2 kHz, acquired at 5 kHz, and displayed and digitized online (Digidata 1440A series A/D board; Molecular Devices, Sunnyvale, CA). Data acquisition and analyses were performed using pClamp10.0 (Molecular Devices), and results were plotted using Origin 8.0 (Origin Lab Corp., North Hampton, MA). All experiments were performed at room temperature (22 ± 1°C).

### Perforated Patch-Clamp Whole-Cell Recordings Using Dissociated VTA Neurons

Both perforated and conventional patch-clamp whole-cell recordings were employed (Yang et al., 2011; Steffensen et al., 2018). The perforated patch recordings were crucial for obtaining stable nicotinic responses from dissociated midbrain neurons, although the precise mechanism involved is still unclear. The pipettes (3–5 MΩ) used for perforated patch recording were filled with intracellular recording solution containing amphotericin B (200 μg/ml). Following tight-seal (2 GΩ) formation, conversion to perforated patch mode typically occurred over 5–15 min, which was monitored by changes in access resistance. An access resistance of less than 60 MΩ was the threshold required to initialize the experimental protocol. Series resistance was not compensated in this study. The data were filtered at 2 kHz, acquired at 5 kHz, and digitized online (Axon Instruments Digidata 1550 series A/D board, Axon Instruments Inc., Union City, CA). All experiments were performed at room temperature (22 ± 1°C). Studies of acutely dissociated neurons were done using cells attached to the cell culture dish, whereas studies of transfected cells expressing human α6-nAChRs were lifted from the dish *via* the recording pipette before initiation of the recording procedure.

### Solutions and Drug Application

#### For Transfected α6-nAChR SH-EP1 Cells

The standard external solution for SH-EP1 cells contained (in mM): 120 NaCl, 5 KCl, 2 MgCl_2_, 2 CaCl_2_, 25D-glucose, and 10 HEPES; pH 7.4 (Tris-base). For traditional whole-cell recording, the pipette solution contained (in mM): 110 Tris-phosphate dibasic, 28 Tris-base, 11 EGTA, 2 MgCl_2_, 0.5 CaCl_2_, and 4 Na-ATP; pH 7.3. In most experiments, nicotine was used as the test agonist and induced whole-cell current responses. Nicotine was quickly perfused onto recorded cells using a computer-controlled U-tube system and the cells were surrounded by applied drugs within 30 ms. The interval between drug applications was optimized to 2 min specifically to ensure the stability of nAChR responsiveness (i.e. no functional rundown).

#### For Dissociated VTA Neurons

The standard external solution contained: (in mM) 140 NaCl, 5 KCl, 2 CaCl_2_, 1 MgCl_2_, 10 HEPES, and 10 glucose, pH adjusted to 7.3 with Tris-base. The amphotericin B-perforated patch-pipette solution used for current-clamp and voltage-clamp recordings contained (in mM): 150 K gluconate, 5 MgCl_2_, and 10 HEPES, pH adjusted to 7.2 with Tris-OH. Amphotericin B was dissolved in DMSO, and the stock solution was diluted with the internal (patch-pipette) solution to a final concentration of 200 μg/ml just before use. The pipette solution for conventional whole-cell recordings contained (in mM): 140 KCl, 4 MgCl2, 0.1 EGTA, 4 ATP, and 10 HEPES, pH adjusted to 7.4 with KOH. Nicotinic agonists were rapidly delivered into the bath medium by a computer-controlled U-tube system, in which the applied drug surrounds the recorded cell in less than 30 ms. The times between drug applications were adjusted specifically to ensure the stability of nAChR responsiveness (absence of functional rundown). The drugs used in the present study were (−) nicotine and cocaine, which were purchased from Sigma-Aldrich, and α-conotoxin MII, which was a gift from Professor Michael McIntosh.

### Expression of Human Neuronal α6N/α3Cβ2β3 and α6M211L/α3IC nAChRs in *Xenopus* Oocytes and Two-Electrode Voltage-Clamp Recording

#### Molecular Constructs

The human α6N/α3C chimera was the same as that used for the SH-EP1 transfection, but in an oocyte expression vector, pGEMHE. Human α6_M211L_/α3_IC_ chimera was constructed according to the sequence (α6_211L_/α3_cyt_) reported by Ley et al. (Ley et al., 2014) from Lindstrom’s lab using the Fast cloning and Quikchange methods (Li et al., 2012). Human β2 and β3 wild-type sequences are found in the oocyte expression vector, pSGEM. The cRNAs encoding these subunits were transcribed by T7 RNA polymerase (New England Biolab, Ipswich, MA, USA) using the standard *in vitro* transcription protocols.

#### Oocyte Preparation and Injection

Oocytes were harvested from female *Xenopus laevis* (Xenopus I, Ann Arbor, MI, USA) as previously described (Xu et al., 2016) by the IACUC-approved protocol of *Xenopus* Care and Use. The stage VI oocytes were selected and incubated at 16°C before injection. Micropipettes for injection were pulled from borosilicate glass (Drummond Scientific, Broomall, PA, USA) on a Sutter P97 horizontal puller, and the tips were cut with forceps to ≈40 μm in diameter. The cRNA mixture with 1:1:1 ratio (α6/α3:α2:α3) was drawn up into the micropipette and injected into oocytes with a Nanoject microinjection system (Drummond Scientific) at a total volume of ~60 nl.

#### Two-Electrode Voltage Clamp

Three to five days after injection, an oocyte expressing α6*-nAChRs was placed in a home-made small volume chamber and continuously perfused with oocyte Ringer’s solution (OR2), which consisted of (in mM) 92.5 NaCl, 2.5 KCl, 1 MgCl2, CaCl2, and 5 HEPES; pH 7.5. The chamber was grounded through an agar salt bridge to avoid drug influence on junction potential between grounding silver wire and solution. The oocytes were voltage-clamped at −70 mV to measure acetylcholine-induced currents using a GeneClamp 500B (Axon Instruments, Foster City, CA, USA). The current signal was low-pass filtered at 50 Hz with the built-in 4-pole low-pass Bessel filter in the GeneClamp 500B and digitized at 100 Hz with a Digidata1440a and pClamp 10 software (Axon Instruments).

#### Homology Modeling and Ligand Docking

Homology modeling of the human α6_M211L_α3_IC_β2β3 receptor construct was performed using ICM Pro 1.7. The crystal structure of human α4β2 (with pdbID of 5kxi) was used as the structural template for this receptor. The α6_M211L_α3_IC_ sequence was aligned with the α4 sequence, and β3 sequence was aligned with the β2 sequence using Clustal Omega. The excess unaligned sequences from N-terminus, C-terminus, and intracellular loop were removed. For the α6_M211L_α3_IC_ subunit used in the model, the majority of α3 intracellular loops were removed (with 17 residues remaining). This chimeric α subunit was modeled using chain A and chain D as structural templates, whereas β3 was modeled with chain C as the template. The pentameric model of α6_M211L_α3_IC_β2β3 was created by putting these three chain models and original β2 crystal structures (chains B and E) together. The new model was further optimized and converted to an ICM object using MolMechanics. The molecular structure of cocaine was obtained from the publicly available crystallographic data corresponding to pdbID of 1q72. Potential ligand-binding domains were identified in the model of whole receptor pentameric structure using “Identify Binding Sites.” In addition to the identification of the orthosteric binding sites located along the subunit interfaces, five potential binding sites in the coupling region between amino-terminal domains and transmembrane domains in all five subunit interfaces were identified. The noncompetitive nature of the cocaine-mediated inhibitory effect observed here suggests that its binding site is different from the traditional orthosteric binding region. Since the inhibitory effect is α6 specific, we selected one potential binding site in the coupling region and the interface between chain D (α6) and chain E (β2) for cocaine docking. This is also consistent with the observation that antagonism is not use-dependent as well as that the binding site is not located in the channel domain. The docking conformation with the lowest energy was used for presentation in the Discovery Studio Visualizer 4.0.

### Data Analysis and Statistics

Both peak and steady-state components of inward currents were measured. For dissociated VTA neurons, sIPSCs were analyzed with Clampfit 10.6 software (MDS Analytical Technologies) as described previously (Gao et al., 2010; Yang et al., 2011). In brief, the sIPSCs were screened automatically using a template with an amplitude threshold of 5 pA. These were visually accepted or rejected based on the rise and decay times. More than 95% of the sIPSCs that were visually accepted were screened using a suitable template, and values of *p* < 0.05 were considered significant. Data are presented as means ± standard errors. Statistical analysis was performed using paired *t*-tests when evaluating data obtained from a single cell and values of *p* < 0.05 were considered significant. One-way ANOVA was used. Curve fitting for agonist and antagonist concentration-response data was performed (Origin 8.0 software; Origin Lab Corp.) using the logistic equation to provide fits for maximal and minimal responses, EC_50_ or IC_50_ values, and Hill coefficients.

## Results

### Cocaine Reduces α6*-nAChR-Mediated Whole-Cell Currents in SH-EP1 Cells

**Figure 1:**
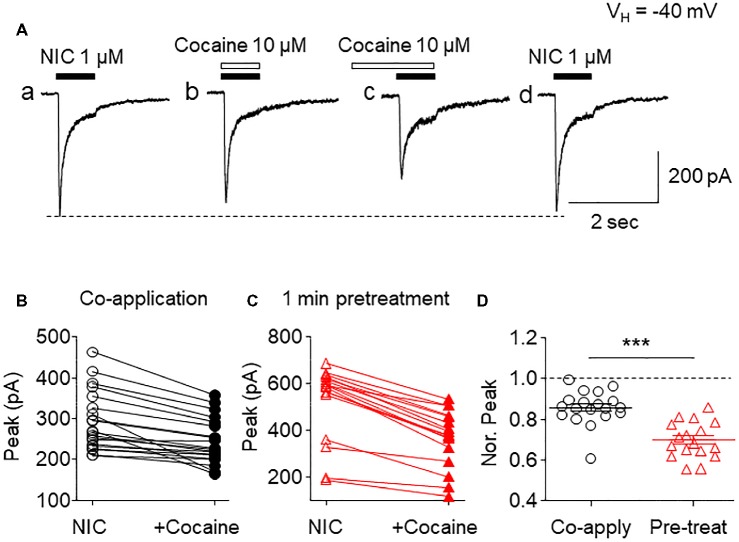
Effects of acute cocaine on α6*-nAChR-mediated whole-cell currents. **(A)** Representative traces of nicotine (NIC)-induced inward currents before and after cocaine exposure. The effects of cocaine on NIC-induced currents (peak amplitude) were compared when cocaine was co-applied with NIC **(B)** or with 1-min cocaine pre-application **(C)**. **(D)** Statistical comparison of the inhibitory rate of cocaine on NIC-induced current (normalized peak amplitude) between co-application and pretreatment of cocaine. ***p < 0.001.

To determine if cocaine affected α6*-nAChR function in SH-EP1 cells, we applied patch-clamp lifted whole-cell recording (Yu et al., 2009) combined with U-tube fast drug application. At a holding potential of −40 mV (close to the resting membrane potential of SH-EP1 cells), bath application of 1 μM nicotine (close to nicotine EC_50_ concentration) induced an inward current (Figure 1Aa). Co-application of 1 μM nicotine and 10 μM cocaine reduced α6*-nAChR-mediated current (Figure 1Ab). Following pretreatment (1 min), cocaine showed a greater inhibitory effect on inward current compared to 10 μM cocaine co-application with nicotine (Figure 1Ac), and following washout of cocaine for 2 min, the inhibitory effect by cocaine was completely recovered (Figure 1Ad). These effects of cocaine are well represented in the pooled data (Figures 1B,C). Statistical analysis showed that the peak amplitude of 1 μM nicotine-induced currents was reduced to 84.2 ± 2.4% (*n* = 20, *p* < 0.01, paired samples *t*-test) by cocaine and nicotine co-application and to 69.7 ± 2.2% (*n* = 17, *p* < 0.001, paired samples *t*-test) by 1 min cocaine pretreatment, respectively. The difference between pretreatment and without pretreatment was statistically significant (unpaired *t*-test analysis shows co-application vs. pretreatment, *p* < 0.001, Figure 1D). These results suggest that acute cocaine reversibly inhibits α6*-nAChR-mediated currents. Since pretreatment of cocaine shows greater inhibition than co-application of cocaine with nicotine, all following experiments were performed with 1 min cocaine pretreatment.

### Cocaine Inhibits α6-nAChR-Mediated Currents in a Concentration-Dependent Manner

**Figure 2:**
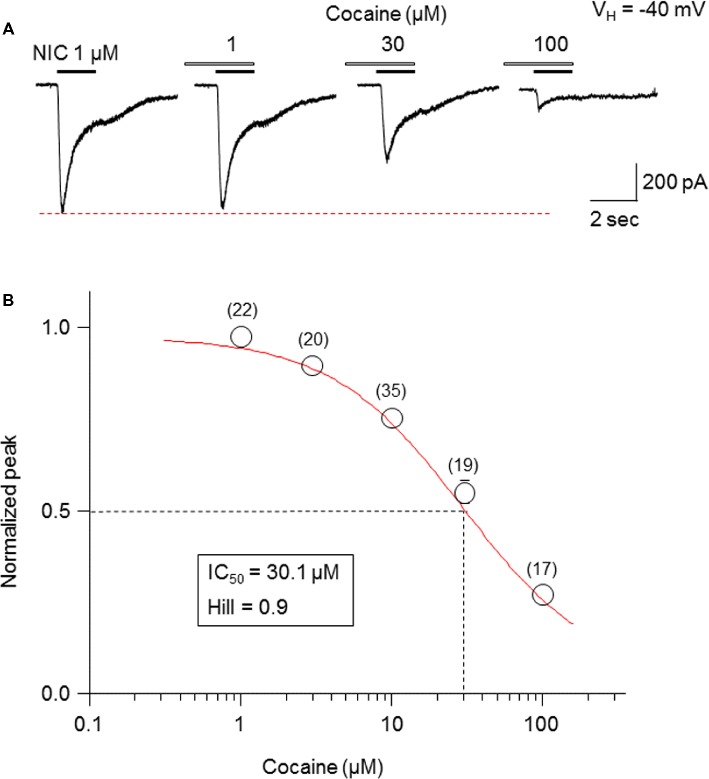
Cocaine inhibits α6*-nAChR-mediated currents in a concentration-dependent manner. **(A)** Representative traces showing the inhibition of NIC-induced currents with different concentrations of cocaine. **(B)** The concentration-inhibition relationship curve from 17 to 35 cells tested (indicated above each symbol) showed a sigmoidal shaped, inhibitory curve of cocaine on NIC response (peak amplitude).

Pretreatment (1 min) with cocaine at different concentrations (1-100 μM) during 1-second nicotine (1 μM) exposure showed a concentration-dependent inhibition in the peak current amplitudes (Figure 2A). The fitting results of concentration-inhibition curves (Figure 2B) showed an IC_50_ value for cocaine-mediated inhibition of whole-cell peak current amplitude of 30.1 μM and Hill coefficient = 0.9.

### Kinetic Analysis of Cocaine-Induced Inhibition in α6*-nAChR-Mediated Currents

**Figure 3:**
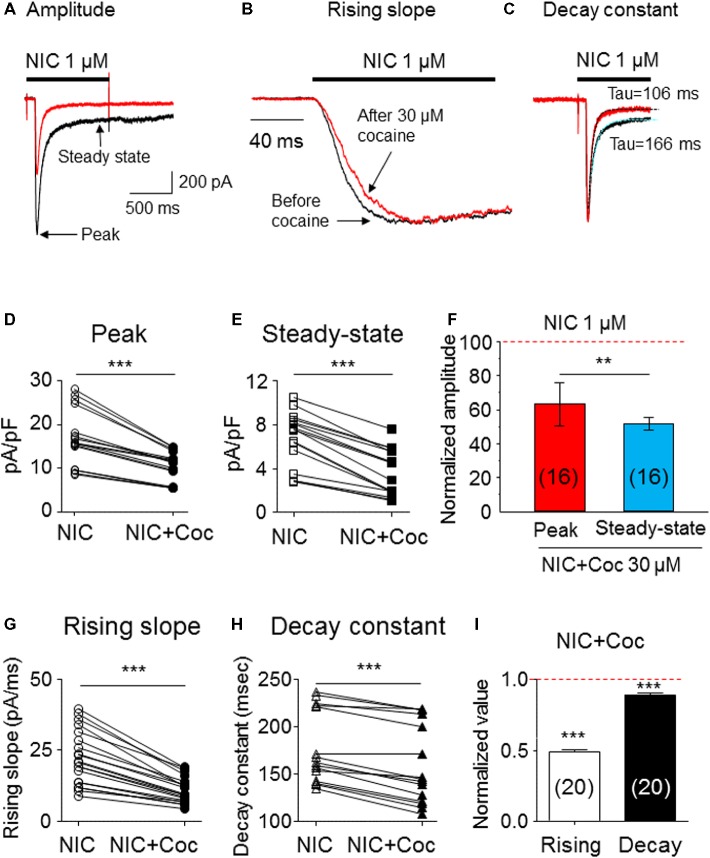
Kinetic analysis of cocaine’s effect on α6*-nAChR-mediated currents. **(A)** Superimposed whole-cell current traces before (black) and after (red) cocaine exposure showing a reduced peak and steady-state components. **(B)** Normalized peak current and expanded time scale to show the rising slope before (black) and after (red) cocaine exposure. **(C)** Single exponential fitting showed the whole-cell current decay time constant during nicotine exposure before (black) and after (red) cocaine exposure. Statistical comparison of the peak **(D)** and steady-state **(E)** components before and after cocaine exposure showed that a cocaine-induced reduction of either peak or steady-state components was highly significant, and the inhibitory effect on steady state was greater **(F)**. **(G)** Cocaine significantly reduced whole-cell current rising slope. **(H)** Cocaine significantly accelerated whole-cell current decay constant. **(I)** The representative inhibitory ratio of cocaine on a whole-cell current rising slope and decay constant. The horizontal dashed lines in F and I indicate the level of nicotine-induced whole-cell current before cocaine exposure, which was normalized to 1.0. **p < 0.01 and ***p < 0.001.

We examined kinetic changes of α6*-nAChR-mediated whole-cell current after acute exposure to 30 μM cocaine. As shown in Figure 3, cocaine reduced 1 μM nicotine-induced whole-cell current amplitude (Figure 3A), slowed current rising slope (Figure 3B), and accelerated decay rate (Figure 3C). Statistical analysis demonstrated that after 30 μM cocaine exposure (with 1-min pretreatment), whole-cell current density of the peak component was reduced from 17.1 ± 1.6 to 10.5 ± 0.9 pA/pF [paired *t*-test, *p* < 0.001, *n* = 16 (Figure 3D)], and the steady-state component was reduced from 6.7 ± 0.6 to 3.7 ± 0.5 pA/pF [paired *t*-test *p* < 0.001, *n* = 16 (Figure 3E)]. Comparison of the inhibitory ratio by cocaine on peak (62.6 ± 1.8%) and steady-state (51.8 ± 3.7%) components showed a highly significant difference [unpaired *t*-test, *p* < 0.001 (Figure 3F)]. In 20 cells tested, 30 μM cocaine slowed α6*-nAChR channel activation represented as a reduced current rising slope [from −22.5 ± 2.1 to −10.9 ± 1.0 pA/ms, paired *t*-test *p* < 0.001, *n* = 20 (Figure 3G)] and accelerated α6*-nAChR channel inactivation measure by current decay constant [Tau numbers from 212.4 ± 15.8 to 194.2 ± 15.7 ms, paired *t*-test *p* < 0.01, *n* = 20 (Figure 3H)]. Therefore, the inhibitory ratio by 30 μM cocaine for channel activation was 50.8 ± 1.5% [paired *t*-test *p* < 0.001 (Figure 3I)] and for the channel, inactivation was 9.5 ± 1.6% [paired *t*-test *p* < 0.01 (Figure 3I)]. These results suggest that acute cocaine inhibits nicotine-induced currents through both channel activation and inactivation phases.

### Cocaine Inhibits α6*-nAChR-Mediated Currents Induced by Different NIC Concentrations

**Figure 4:**
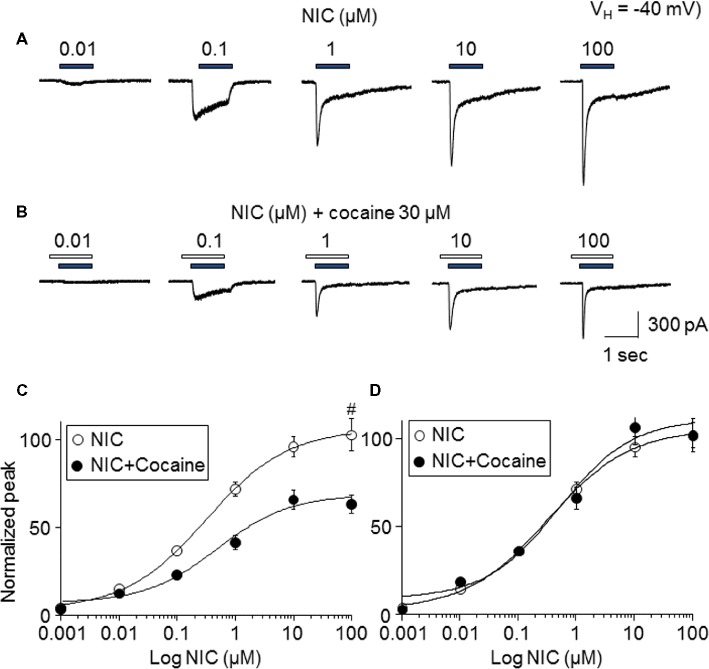
Effects of cocaine on different concentration NIC-induced currents. **(A)** Typical traces showed NIC alone with different concentration-induced inward currents. **(B)** Typical traces showed 30 μM cocaine (with 1 min pretreatment) plus NIC with different NIC concentration-induced inward currents. Panels **(A,B)** were recorded from the same cell. **(C)** Sigmoidal NIC concentration-response curves without (open circle) and with (filled circle) 30 μM cocaine. All symbols were normalized to NIC 100 μM-induced current (indicated as #). **(D)** Sigmoidal NIC concentration-response curves without (open circle) and with (filled circle) 30 μM cocaine. Two group data were normalized to their own maximal NIC concentration (100 μM)-induced current.

To determine the mechanism of how cocaine affects nicotine-induced responses, the nicotine concentration-response relationship curves with or without 30 μM cocaine (with 1 min pretreatment) were compared. As shown in Figure 4, nicotine-induced inward currents increased with increasing NIC concentrations (Figure 4A), forming a sigmoidal-shaped concentration-relationship curve (Figure 4C, open symbols). In the presence of 30 μM cocaine, the different concentrations of nicotine-induced currents were reduced (Figure 4B) compared to nicotine exposure alone (Figure 4C, filled symbols). Paired *t*-test analysis for each nicotine concentration pair with 30 μM cocaine showed highly significant differences between nicotine alone and nicotine plus cocaine at all nicotine concentrations between 0.01 and 100 μM. Using sigmoidal fitting, the EC_50_ values of NIC-induced whole-cell peak current was 0.42 μM and Hill coefficient was 0.8, while with 30 μM cocaine, the EC_50_ values of nicotine plus cocaine-induced whole-cell peak current was 0.45 μM and Hill coefficient was 0.9; the EC_50_ values of nicotine with and without cocaine were not significantly different (unpaired *t*-test). Collectively, cocaine reduces the maximal NIC response of the NIC concentration-response curve (Figure 4C) but does not change the NIC EC_50_ value (Figure 4D), suggesting a noncompetitive inhibitory mechanism.

### Cocaine Inhibitory Effect on α6-nAChR-Mediated Current Is Not Use-Dependent

**Figure 5:**
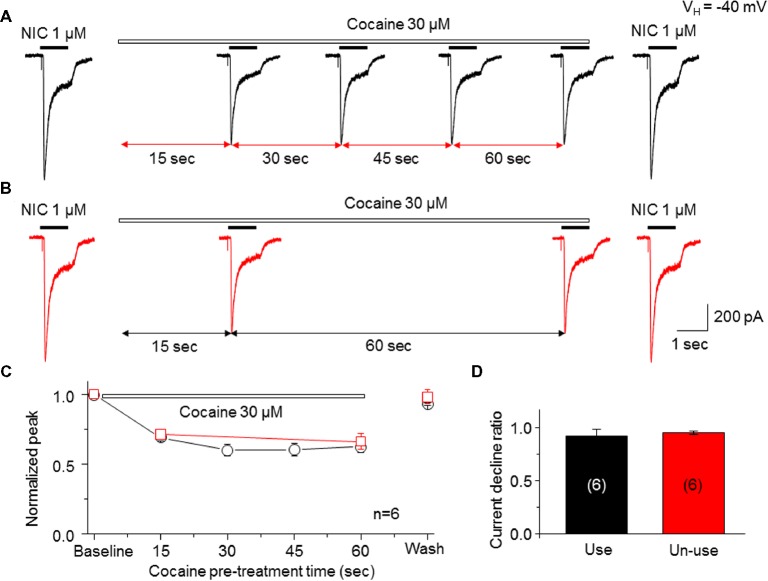
Cocaine inhibits an α6*-nAChR-mediated current in a nonuse-dependent manner. **(A)** NIC use protocol includes repetitive applications of NIC four times with 15-s interval in the presence of cocaine. **(B)** NIC nonuse protocol includes repetitive applications of NIC two times with 60-s interval in the presence of cocaine. **(C)** Time-dependent decline of NIC current with four applications (black) and with two applications (red) in the presence of cocaine. **(D)** Comparison of the current declined ratio between the “use” (4th NIC response/1st NIC response, *n* = 6) and the “nonuse” (2nd NIC response/1st NIC response, *n* = 6) protocols showed no significant difference.

To determine whether the open channel block mechanism underlies the cocaine-induced noncompetitive inhibition in the α6*-nAChR-mediated current, we examined a use-dependent feature of cocaine inhibition. We compared the current decline ratio with four repetitive applications at 15-s interval of nicotine (use-dependent) or only two applications of nicotine given at a 60-s interval (nonuse-dependent) when cocaine (30 μM) was persistently present. As shown in Figure 5A, repetitive applications (four times at 15-s interval) of nicotine (1 μM for 1 s) with the continuous presence of 30 μM cocaine led to nearly unvarying suppression in the nicotine-induced currents, and following washout for 2 min, the nicotine response completely recovered. Under the same experimental conditions, repetitive applications of nicotine (1 μM for 1 s) twice (with 60 s interval) in the continuous presence of 30 μM cocaine led to a similar suppression in the NIC-induced currents (Figure 5B). Again, following 2-min washout, the nicotine response completely recovered. In six cells tested, the nicotine response declined to the same level using these two experimental protocols when cocaine was persistently present (Figure 5C). Comparison of current declined ratio between the use (4th nicotine response/1st nicotine response) and nonuse (2nd nicotine response/1st nicotine response) protocols showed no significant difference (Figure 5D), suggesting that cocaine inhibition of α6*-nAChR-mediated current is not use-dependent.

### Cocaine Inhibition of α6-nAChR-Mediated Current Is Not Mediated Through Intracellular Targets

**Figure 6:**
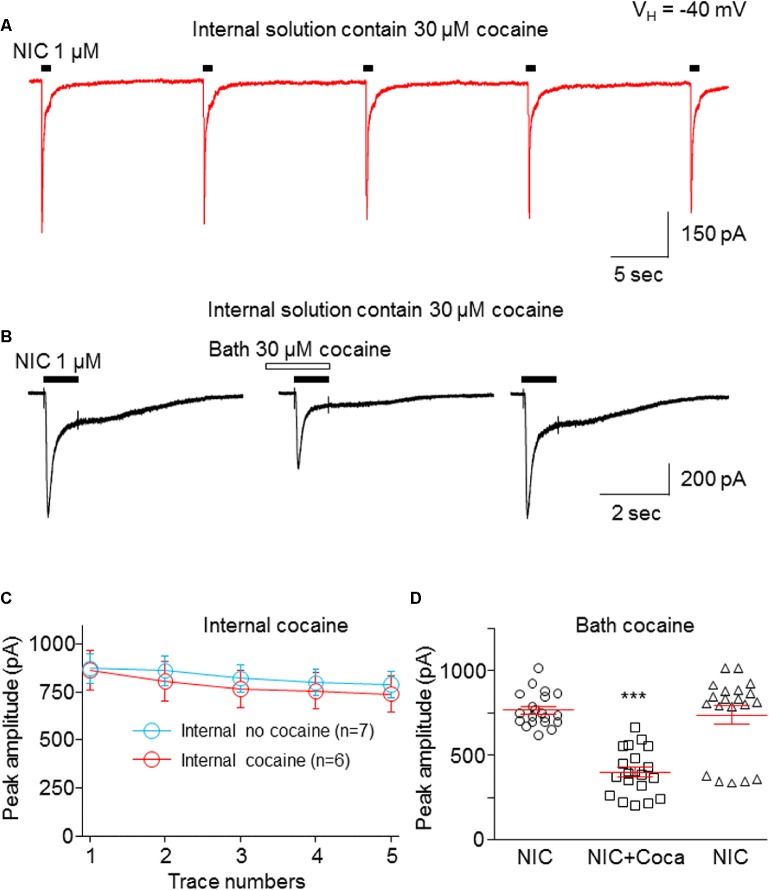
Cocaine inhibits α6*-nAChR-mediated current through the extracellular site. **(A)** Cocaine (30 μM) was added to pipette solution. After the formation of whole-cell recording configuration for 3–5 min, 1 μM NIC was repetitively applied (1 s NIC exposure repeated five times at 15-s interval). The same experimental protocol was also applied without internal cocaine as a control (see Figure 7C). **(B)** After recording from the protocol in **(A)**, bath-applied cocaine (30 μM with 1 min pretreatment) to the same cell showed an inhibitory effect. **(C)** Comparison of NIC responses with and without internal cocaine. **(D)** Statistical comparison of cocaine’s effect *via* bath application. ***p < 0.001.

To test whether the cocaine-induced inhibition in α6*-nAChR-mediated current occurs at an extracellular or intracellular target, we added 30 μM cocaine into the recording electrode. Following the formation of the whole-cell recording configuration for 3–5 min to allow cocaine to infuse into the cell, we examined repetitive nicotine-induced current response (1 μM for 1 s, repeated five times at 15-s interval) under this intracellular cocaine condition. Figure 6 demonstrates that intracellular administration of 30 μM cocaine produced five stable and unchanged repetitive nicotine-induced current responses (Figure 6A). While there was no change in the nicotine response in the presence of intracellular cocaine, bath application of 30 μM cocaine inhibited the nicotine response (Figure 6B). Figure 6C summarizes the pooled data, in which the nicotine-induced current showed no difference with versus without internal cocaine. Figure 6D summarizes the data showing that the intracellular cocaine did not alter nicotine response, whereas the bath application of cocaine inhibited nicotine response. These results suggest that cocaine inhibits α6*-nAChR-mediated current through extracellular sites.

### Effects of Cocaine on α6N/α3Cβ2β3- and α6_M211L_α3_IC_ β2β3-nAChRs Expressed in *Xenopus* Oocytes

**Figure 7:**
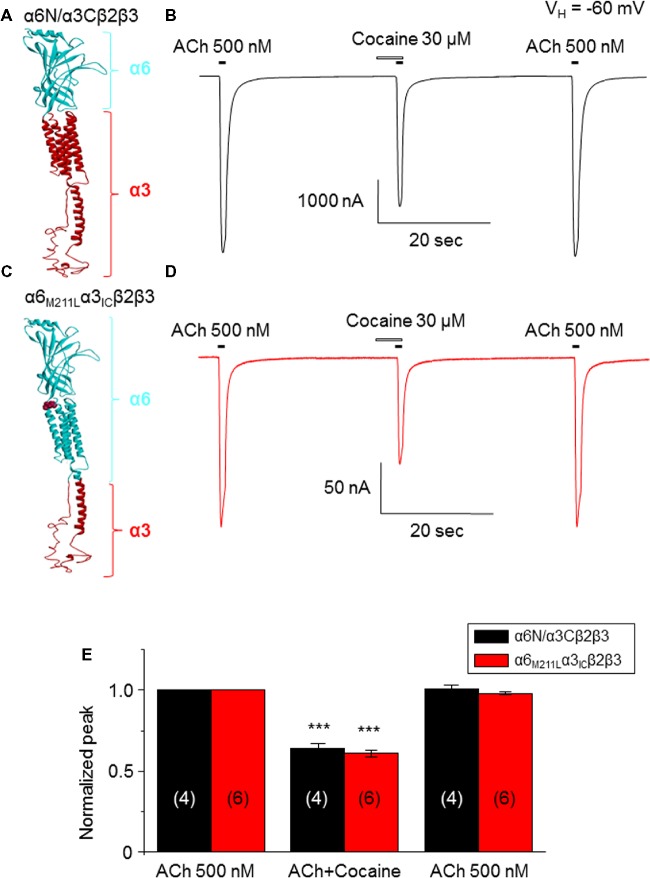
Cocaine inhibits two types of α6*-nAChR-mediated currents in *Xenopus* oocytes. **(A)** 3D structure of the α6Nα3C subunit, which is similar to the heterologously transfected α6/α3 chimera in human EH-EP1 cells. **(B)** Cocaine inhibited ACh-induced current in the α6N/α3Cβ2β3-nAChR. **(C)** 3D structure of the α6_M211F_α3_IC_ subunit, which contains more content (N-terminal and four transmembrane domains) of α6 subunit. **(D)** Acute cocaine effect on the α6_M211L_α3_IC_β2β3 nAChR. **(E)** Statistical comparison of cocaine’s effect on α6Nα3Cβ2β3 nAChR- and α6_M211F_α3_IC_β2β3 nAChR-mediated currents, with the similar inhibitory rate. ***p < 0.001 (paired *t*-test) when cocaine effect is compared to the control.

**Figure 8:**
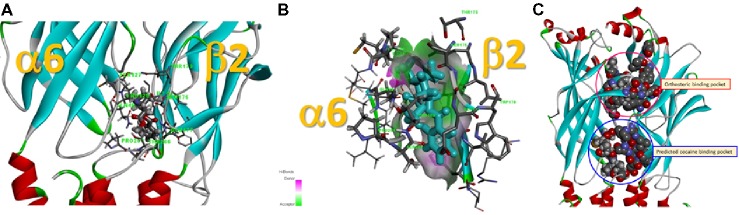
Potential cocaine binding site in the α6_M211L_α3_IC_β2β3-nAChR. **(A)** Cocaine docked to a potential binding pocket identified by the software in the subunit interface between α6_M211L_α3_IC_ and β2 subunits and in the coupling region between N-terminal domain and a transmembrane domain. **(B)** A closer look at the potential cocaine interactions with the receptor. **(C)** Comparison of the binding sites between nicotine (orthosteric binding pocket) and cocaine (predicted cocaine binding pocket).

Data presented thus far demonstrate that acute exposure to cocaine inhibits an α6*-nAChR-mediated current in human SH-EP1 cells. Since simply transfecting WT nAChR α6 with any other β subunits into the expression system does not result in functional receptors, we used an α6N/α3Cβ2β3 combination and transfected it into SH-EP1 cells. Our previous publications show these transfected cells exhibit excellent function with the features consistent with α6*-nAChR pharmacology and physiology (Damaj et al., 1999; Francis et al., 2000). To explore the possibility that cocaine acts on the α6 subunit, we followed a recent report (Ley et al., 2014) to create a mutant α6_M211L_/α3_IC_. The receptor N terminal and four transmembrane domains are formed from α6 with a single point mutation (e.g., M211 L), and only the second intracellular loop is from α3. With this subunit combination, the receptor contains primarily α6 content. We compared cocaine effects on the α6N/α3Cβ2β3-nAChR (Figure 7A), and the α6_M211L_/α3_IC_ β2β3-nAChR (Figure 7C) expressed in *Xenopus* oocytes using the two-electrode voltage-clamp recording. The results demonstrated that cocaine (30 μM with 1 min pretreatment) inhibited 500 nM ACh (close to ACh EC_50_)-induced currents with a similar inhibitory rate in these two variants of α6*-nAChRs (Figures 7B,D,E). These results suggest that cocaine likely acts on the α6 subunit to inhibit α6*-nAChR-mediated current. To determine the potential binding site of cocaine in the nicotinic receptor, we docked cocaine to the homology model of α6_M211L_α3_IC_β2β3 nAChR with ICM Pro 1.7 software (see Methods for detail). The docking conformation with the lowest energy was used for presentation. As shown in Figure 8, cocaine docked to a potential binding pocket in the subunit interface between α6_M211L_α3_IC_ and β2 subunits (Figure 8A) and the coupling region between the N-terminal domain and a transmembrane domain (Figure 8B). Figure 8C compares the binding sites between nicotine (top) and cocaine (bottom).

### Cocaine Inhibits Natural α6*-nAChRs on VTA GABAergic Boutons on DA Neurons

**Figure 9:**
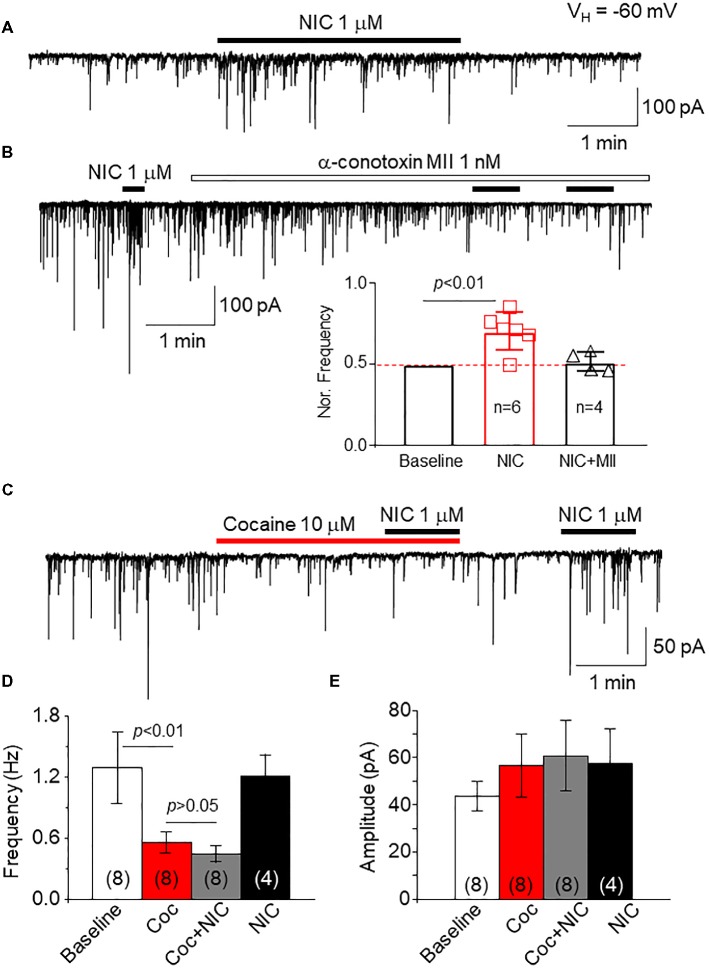
Cocaine inhibits natural α6*-nAChRs expressed on GABAergic boutons on VTA DA neurons. **(A)** In mechanically dissociated VTA GABA neurons, patch-clamp whole-cell recording in voltage-clamp mode demonstrated sIPSCs and bath application of 1 μM NIC enhanced sIPSC frequency. **(B)** Pretreatment with the α6*-nAChR selective antagonist, α-conotoxin MII (1 nM for 5 min) prevented NIC-induced effect, suggesting that α6*-nAChRs mediate the NIC effect. **(C)** Bath application of cocaine (10 μM) reduced the sIPSC frequency, and in the presence of cocaine, NIC faired to enhance sIPSCs. After washout of cocaine for 1 min, NIC enhanced sIPSCs. Statistical analysis of the effects of cocaine on sIPSCs and NIC-induced effect demonstrated that compared to a baseline level, cocaine significantly reduced the sIPSC frequency, and in the presence of cocaine, NIC faired to enhance sIPSC frequency **(D)**. However, cocaine did not affect sIPSC amplitude **(E)**.

To examine the effects of cocaine on natural α6*-nAChRs, we used a mechanically dissociated VTA DA neuron model, in which we have demonstrated that functional α6*-nAChRs are expressed on GABAergic presynaptic boutons synapsed onto DA neurons (Yang et al., 2011). As shown in Figure 9A, we recorded sIPSCs in isolated single DA neurons under voltage-clamp recording mode, and bath application of 1 μM nicotine increases sIPSCs frequency. However, after pretreatment for 5 min with an α6*-nAChR-selective antagonist, α-Ctx MII (1 nM), the nicotine-induced enhancement of sIPSCs was abolished (Figure 9B), suggesting that the NIC-induced effect is mediated through presynaptic α6*-nAChRs. By using this cell model, bath application of cocaine (10 μM) suppressed the sIPSC frequency but not amplitude, and under this condition in the presence of cocaine, bath application of NIC failed to enhance sIPSCs (Figure 9C). In four cells tested, bath application of nicotine enhanced sIPSC frequency again following a 5-min washout of cocaine. Statistical analysis showed that cocaine inhibited sIPSC frequency and prevented nicotine-induced enhancement of sIPSCs (Figure 9D), but cocaine did not show significant effects on sIPSC amplitude (Figure 9E). These results suggest that cocaine can inhibit α6*-nAChRs naturally expressed on GABAergic presynaptic boutons synapsing on VTA DA neurons.

## Discussion

This study suggests that cocaine directly inhibits α6*-nAChR function, which is a novel finding. By using heterologously expressed α6*-nAChRs in human SH-EP1 cells, we demonstrate that bath-applied cocaine inhibits an α6*-nAChRs-mediated whole-cell current in a concentration-dependent manner with an IC_50_ value of 30 μM. Kinetic analysis shows that cocaine slows nicotine channel activation and accelerates α6*-nAChR desensitization, which together reduces the amplitude of the nicotine-induced current. Cocaine inhibits α6*-nAChRs through an extracellular site in a noncompetitive way, but not in a use-dependent way. In *Xenopus* oocytes, cocaine equally inhibits α6Nα3Cβ2β3- and α6_M211L_α3_IC_β2β3-mediated currents, suggesting that the α6 subunit is likely the target for cocaine inhibition, which is further confirmed by docked prediction modeling. In mechanically dissociated VTA DA neurons, cocaine inhibits α6*-nAChRs-mediated enhancement of sIPSC frequency, suggesting that cocaine can inhibit natural presynaptic α6*-nAChR function. Together, this study provides the first evidence that cocaine directly inhibits α6*-nAChR function, suggesting that α6*-nAChRs may play an important role in cocaine reward and dependence.

### Heterologously Expressed α6*-nAChR Is a Good Cell Model to Test Cocaine’s Effects

nAChRs containing α6 subunits are not widely expressed in the brain, but are prevalent in midbrain dopamine neurons. Thus, α6*-nAChRs are associated with pleasure, reward, and mood control (Klink et al., 2001; Azam et al., 2002), suggesting that α6*-nAChRs may play critical roles in drug reward and addiction (Shytle et al., 2002). However, the functional and pharmacological properties of α6*-nAChRs are largely unknown because of two major reasons: (1) there are no selective α6*-nAChR agonists and (2) examining the function of heterologous expression of nAChR α6 subunit with other β subunits has proven challenging. Previous studies showed that heterologous expression of α6 in any combination involving β2 subunits that result in functional ion channels is extremely difficult to study at a functional level. However, chimeric subunits where the N-terminal domain of α6 is fused to the transmembrane domain of α3 or α4 (α6/α3 or α6/α4) were found to produce functional receptors with β2 or β4 subunits (Kuryatov et al., 2000). In the present study, we used an established functional α6*-nAChR developed through co-transfection of α6/α3 chimera and β2, β3 subunits into a human SH-EP1 cell line (Breining et al., 2012). We recently profiled the functional and pharmacological features of this α6*-nAChR and showed that this transfected α6*-nAChR exhibits excellent function and is consistent with α6*-nAChR pharmacology (Chen et al., 2018), yielding an effective cell model to evaluate the effects of cocaine on α6*-nAChR function.

### Cocaine Inhibits the α6*-nAChR Function

It has been reported that cocaine inhibits heterologously expressed nAChR function in *Xenopus* oocyte. For example, Damaj et al. reported that cocaine inhibits heterologously expressed nAChR-mediated current in a concentration-dependent manner with the IC_50_ range of 4.4–6.9 μM for α4β2-nAChRs and 22–42.3 μM for α3β2-nAChRs (Damaj et al., 1999). They found that cocaine-induced inhibition in nAChR function is through a noncompetitive mechanism (Damaj et al., 1999). Francis et al. systematically compared cocaine-induced inhibition in different subtypes of nAChRs expressed in oocytes and found that α4β4-nAChRs exhibited the highest sensitivity with the IC_50_ of 2 μM and α3β2 showed the lowest sensitivity with the IC_50_ of 60 μM (Francis et al., 2000). In addition to these expressed nAChRs, Acevedo-Rodriguez et al. found that cocaine also inhibits native nAChRs (β2-containing) in midbrain dopaminergic neurons with the IC_50_ of 19.8 μM (Acevedo-Rodriguez et al., 2014). Collectively, these previous data suggest that cocaine serves as a low-affinity antagonist on different subtypes of nAChRs with varying affinities. However, thus far, whether cocaine modulates α6*-nAChR function is unknown. The present study provides the first evidence that within a physiologically relevant concentration range, cocaine inhibits α6*-nAChR function in a concentration-dependent manner with an IC_50_ value of 30 μM. Within the nicotine concentration-response curve, different nicotine concentrations with 30 μM cocaine reduce the maximal nicotine concentration-induced current without changing the nicotine EC_50_ value, suggesting a noncompetitive inhibition. Our data are consistent with previous reports that also expressed nAChRs in *Xenopus* oocytes and demonstrated that cocaine inhibits human α6*-nAChRs heterologously expressed in human SH-EP1 cells. In addition to using expressed α6*-nAChRs, we also tested the effects of cocaine on natural α6*-nAChRs located on GABAergic presynaptic boutons/terminals on VTA DA neurons (Yang et al., 2011). Thus far, it is difficult to record α6*-nAChR-mediated whole-cell current in wild-type rodents unless gained α6*-nAChR transgenic mice are used (Engle et al., 2013). Even in the gained α6 subunit transgenic mice, the α6 subunit is likely assembled with an α4 subunit to form the α4α6-containing nAChRs (Drenan et al., 2008). Previously, we found functional α6*-nAChRs located on presynaptic GABAergic boutons/terminals on VTA DA neurons (Yang et al., 2011) and also on GABA neurons (Steffensen et al., 2018). In the present study, we tested the effects of cocaine on the function of these presynaptic α6*-nAChRs. As shown in Figure 9, the presynaptic α6*-nAChRs can be activated by 1 μM nicotine, which can then be abolished by 1 nM a-Ctx MII. By using this cell model, we showed that 10 μM cocaine abolished the nicotine (1 μM)-induced increase in sIPSC frequency. These results suggest that cocaine inhibits presynaptic α6*-nAChR function with a higher potency compared to expressed α6*-nAChR. Interestingly, Acevedo-Rodriguez et al. found that cocaine-induced inhibition of presynaptic nAChR-mediated Ca^2+^ release is more sensitive than that of postsynaptic nAChRs located on the cell body (Acevedo-Rodriguez et al., 2014).

### Mechanisms of Cocaine-Induced Inhibition in α6*-nAChRs

In this study, we designed a series of experiments to explore the possible mechanisms of cocaine-induced inhibition in α6*-nAChR. First, we compared nicotine concentration-response curves with or without 30 μM cocaine. We showed that in the presence of cocaine, the nicotine concentration-response curve was shifted downward without changing the EC_50_ value, suggesting noncompetitive inhibition, which is consistent with the cocaine-induced inhibition in other subtypes of nAChRs (Damaj et al., 1999; Francis et al., 2000). Second, we performed the use-dependent experiment and found that cocaine-induced inhibition was not use-dependent, suggesting that cocaine-induced inhibition in α6*-nAChR may not be mediated through an open channel block mechanism. Third, through the kinetic analysis, we found that cocaine slowed nicotine channel opening kinetics and accelerated α6*-nAChR desensitization during nicotine exposure. Finally, we applied cocaine intracellularly through the recording electrode and found that intracellular cocaine did not affect α6*-nAChR function, suggesting that cocaine acts on an extracellularly located site.

### Significances and Limitations

We have provided evidence that cocaine inhibits heterologously expressed and natural α6*-nAChR function. Since these receptors are localized in brain regions associated with pleasure, reward, drug reinforcement, drug dependence, control of mood and emotion, and control of attention, α6*-nAChRs could play important roles in the modulation of these functions. Especially, midbrain DA neurons and associated circuits play a major role in drug reward and dependence, and α6*-nAChRs play a critical role in modulation of mesolimbic function. Therefore, the finding that cocaine directly inhibits α6*-nAChRs is highly significant toward understanding cocaine targets, mechanisms, and therapeutics. For instance, we show that cocaine prevents cholinergic (1 μM nicotine) enhancement of presynaptic sIPSCs on VTA DA neurons, in turn enhancing VTA DA neuron activity. However, since large numbers of α6*-nAChRs are expressed on DA neuronal terminals in the NAc, inhibition of these α6*-nAChRs by cocaine may reduce DA release in NAc. This idea is supported by previous data that cocaine reduced DA release in the NAc (Acevedo-Rodriguez et al., 2014). Our results suggest that α6*-nAChRs in the midbrain may play a critical role in the control of mesolimbic signaling strength during cocaine exposure. Thus, the cocaine-induced modulations on α6*-nAChRs may be involved in cocaine reward and addiction process.

We realize a limitation of this study, as discussed above, is that our heterologously expressed α6*-nAChRs are not wild-type α6 subunits, but are α6 and α3 subunit chimeras. Since no wild-type α6 subunits combined with β subunits can form functional nAChRs, the α6/α3 chimeras with β2β3 formed functional α6*-nAChR is only our choice to evaluate cocaine’s effect on these receptor types. Although we have demonstrated that these expressed α6*-nAChRs are representative of α6*-nAChR function and pharmacology (Chen et al., 2018) and have been used for new drug screening (Breining et al., 2012), this study cannot precisely confirm the acting site of cocaine on α6*-nAChRs. To overcome this weakness, we designed two experiments. First, we confirm that cocaine inhibits α6*-nAChR function through extracellular but not intracellular site since the internal application of cocaine failed to show any inhibitory effect (Figure 4). Second, we examined cocaine’s effects on the α6_M211L_α3_IC_β2β3-nAChR expressed in *Xenopus* oocytes. Since α6_M211L_α3_IC_β2β3-nAChR contains more α6 subunit components including the N-terminal and all four transmembrane domains, but with α3 second internal loop and with a point mutation (e.g., M211 L), we compared cocaine inhibition in α6Nα3Cβ2β3-nAChR- and α6_M211L_α3_IC_β2β3-nAChR-mediated currents. We found that cocaine equally inhibits these two types of α6*-nAChRs (Figure 5). Furthermore, we employed a cocaine docking homology model of α6_M211L_α3_IC_β2β3-nAChR with ICM Pro 1.7 software (see Methods for detail). The docking conformation with the lowest energy was used for presentation. Results show that cocaine docks to a potential binding pocket identified by the software in the subunit interface between α6_M211L_α3_IC_ and β2 subunits and the coupling region between the N-terminal domain and transmembrane domain (Figure 8).

The location of the predicted cocaine binding site by our docking is consistent with a noncompetitive antagonist mechanism. Since the channel activation involves an expansion in the coupling region and extracellular end of the transmembrane domain (Sauguet et al., 2014), cocaine binding in the subunit interface of the nicotinic receptor would prevent the similar expansion and antagonize the receptor. This is in contrast to the cocaine binding to the orthosteric ligand binding pocket found in the crystal structure of AChBP (Hansen and Taylor, 2007). Although they suggested non-alpha subunit interface for the noncompetitive antagonists, the ligands for the allosteric site for benzodiazepines in GABA_A_ receptors (equivalent site for non-alpha subunit interface for nicotinic receptor) has never been found to noncompetitively antagonize the receptor. Thus, the ligand binding to that site could be a negative allosteric modulator. A noncompetitive antagonist binding site could be located within the channel pore. However, nonuse dependence of the cocaine inhibition we observed here does not support that mechanism either. Thus, cocaine likely binds to the coupling region between two subunits and prevents them from moving apart for channel opening. Validation and mapping of the competed binding pocket of cocaine in this channel is awaiting future experiments with single point mutations along with substituted cysteine accessibility methods.

## Author Contributions

DC performed some patch-clamp recordings, analysis and interpretation, and the figures making. FG contributed in acquisition of patch-clamp recording data, analysis and interpretation, and the figures making. XM performed some patch-clamp recordings, analysis and interpretation. JE contributed in SH-EP1 cells cultures. YH and ZM performed some patch-clamp recordings. MG performed some patch-clamp recordings and data analysis. YC contributed in study design, acquisition of the electrophysiology data recorded from oocytes, and writing a part of the manuscript. TD-G contributed in data analysis and assistance of paper writing. JN contributed in study concept and design, and revised manuscript. PW and QS contributed in study design, interpretation of data, and revising the manuscript. JW contributed in study concept and design, acquisition of electrophysiology data, analysis and interpretation, statistical analysis, drafting and revising the manuscript.

### Conflict of Interest Statement

The authors declare that the research was conducted in the absence of any commercial or financial relationships that could be construed as a potential conflict of interest.
